# Applications of Stochastic Process Models to Constructing Predictive Models of Alzheimer’s Disease

**DOI:** 10.1093/geroni/igaa057.844

**Published:** 2020-12-16

**Authors:** Konstantin Arbeev, Olivia Bagley, Arseniy Yashkin, Hongzhe Duan, Igor Akushevich, Svetlana Ukraintseva, Anatoliy Yashin

**Affiliations:** Duke University, Durham, North Carolina, United States

## Abstract

Large-scale population-based data collecting repeated measures of biomarkers, follow-up data on events (incidence of diseases and mortality), and extensive genetic data provide excellent opportunities for applying statistical models for joint analyses of longitudinal dynamics of biomarkers and time-to-event outcomes that allow investigating dynamics of biomarkers and other relevant factors (including genetic) in relation to risks of diseases and death and how this may propagate to the future. Here we applied one such model, the stochastic process model (SPM), to data on longitudinal trajectories of different variables (comorbidity index, body mass index, cognitive scores), other relevant covariates (including genetic factors such as APOE polymorphisms and polygenic scores, PGS), and data on onset of Alzheimer’s disease (AD) in the Health and Retirement Study. We observed that different aging-related characteristics estimated from trajectories of respective variables in SPM are strongly associated with risks of onset of AD and found that these associations differ by sex, APOE status (carriers vs. non-carriers of APOE e4) and by PGS groups. The approach allows modeling and estimating time trends (e.g., by birth cohorts) in relevant dynamic characteristics in relation to the disease onset. These results provide building blocks for constructing the models for forecasting future trends and burden of AD that take into account dynamic relationships between individual trajectories of relevant repeatedly measured characteristics and the risk of the disease. Such models also provide the analytic framework for understanding AD in the context of aging and for finding genetic underpinnings of such links between AD and aging.

